# A sensitive acupuncture needle microsensor for real-time monitoring of nitric oxide in acupoints of rats

**DOI:** 10.1038/s41598-017-06657-3

**Published:** 2017-07-25

**Authors:** Lina Tang, Yutao Li, Hui Xie, Qing Shu, Fan Yang, Yan-ling Liu, Fengxia Liang, Hua Wang, Weihua Huang, Guo-Jun Zhang

**Affiliations:** 1 School of Laboratory Medicine, Hubei University of Chinese Medicine, 1 Huangjia Lake West Road, Wuhan, 430065 China; 2 Department of Acupuncture and Moxibustion, Hubei University of Chinese Medicine, 1 Huangjia Lake West Road, Wuhan, 430065 China; 3 Hubei Provincial Collaborative Innovation Center of Preventive Treatment, 1 Huangjia Lake West Road, Wuhan, 430065 China; 4 Key Laboratory of Analytical Chemistry for Biology and Medicine, Ministry of Education, College of Chemistry and Molecular Sciences, Wuhan University, 4. Bayi Road, Wuhan, 430072 China

## Abstract

This study reports an acupuncture needle modified with an iron-porphyrin functionalized graphene composite (FGPC) for real-time monitoring of nitric oxide (NO) release in acupoints of rats. A gold film was first deposited to the needle surface to enhance the conductivity. The FGPC was prepared via hydrothermal synthesis, and subsequently applied to the tip surface of acupuncture needle by electrochemical deposition method. The functionalized needle enabled a specific and sensitive detection of NO based on the favorably catalytic properties of iron-porphyrin and the excellent conductivity of graphene. Amperometric data showed that the needle achieved not only a low detection limit down to 3.2 nM in PBS solution, but also a satisfactory selectivity. Interestingly, the functionalized needle could be inserted into the acupoints of rats for real-time monitoring of NO *in vivo*. It was found that a remarkable response to NO was respectively obtained in different acupoints when stimulated by _L_-arginine (_L_-Arg), revealing that the release of NO was detectable in acupoints. We expect this work would showcase the applications of acupuncture needle in detecting some important signaling molecules *in vivo*, and exploring the mechanism of acupuncture treatment.

## Introduction

Acupuncture has been employed to treat human disease or to maintain homeostasis for thousands of years^1, 2^. However, the mechanisms of action are still largely unknown. Evidence has been shown regarding neural involvement and nociception^3–8^. It is possible that acupuncture may alter local circulation when the action of acupuncture against pain is taken. Nitric oxide (NO), as an important messenger molecule, is involved in a number of physiological activities (such as neurotransmission, vasodilation, immune responses, and angiogenesis)^9–11^. Since NO is the key regulator of local circulation^12–14^, much attention has been paid to understand how acupuncture regulates the NO levels. Li *et al*.^15^ reported the effect of warm needling on NO levels and found that NO content in blood increased significantly after warm needling. Recently, Tsuchiya *et al*.^16^ developed a method of determining NO generation in blood by using high performance liquid chromatograph and electron spin resonance spectrometer after acupuncture was performed in different acupoints (acupoints: particular bodily locations used by practitioners to elicit therapeutic changes with acupuncture or acupressure). They concluded that acupuncture stimulation enhanced the generation of NO in acupoints and increased local circulation, which could be a modulator of *in vivo* NO levels. Ma *et al*.^17^ detected the skin resistance on acupoints along the meridians (meridians: a network connecting acupoints defined by Traditional Chinese Medicine) in rats to examine the distributions of NO in acupoints. They found that NO content was much greater in acupoints than in brain regions and blood vessels. However, the existing methods for NO detection are based on either *in vitro* analysis (blood), or measurements of the skin stimulus-evoked electrical currents, which can not reflect the body’s instantaneous reaction. As a result, real-time and *in vivo* monitoring of NO in acupoints is of great significance while acupuncture is manipulated on the body.

The current methods for real-time and *in vivo* analysis are mainly dependent on fluorescence-based approaches and electrochemical amperometric methods. Fluorescence microscopy imaging is intuitive and vivid for 3D imaging^18–20^. However, the limits of fluorescence imaging lie in the following aspects. For instance, the temporal resolution is not high enough, and the fluorescent dyes are harmful and undergo photobleaching. Moreover the imaging instruments are usually expensive. Electrochemical methods^21–26^, with the advantages of low cost, ultrafast response and high sensitivity, provide a useful tool for real-time monitoring of biomolecules. However, for real sense of *in vivo* and real-time detection, the electrochemical sensor’s performance in terms of sensitivity and miniaturization must be improved.

Great efforts have been made to ensure that electrochemical sensors have sufficient sensitivity and selectivity to precisely detect NO. Different types of nanomaterials such as carbon nanotubes (CNT)^27–29^, metal nanoparticles^30, 31^ and nanowires^32^, etc, have been employed to develop sensitive NO sensors. Wu *et al*.^33^ reported a multi-walled carbon nanotubes (MWNTs) modified glass carbon electrode (GCE) for electrochemical oxidation of NO. Li *et al*.^32^ fabricated a microelectrode comprising TiC/C nanowire arrays for real-time monitoring of NO from the cultured endothelial cells (ECs). Recently, graphene^34, 35^, with its unique properties, has emerged as a rising star material. It greatly promotes the development of electrochemistry, and has shown a great capacity in accelerating the electron transfer. Moreover, some molecules like porphyrin^36, 37^ or hemin^38, 39^ have also been employed to combine with the above-mentioned nanomaterials to further enhance the selectivity and sensitivity. For example, Liu *et al*.^40^ has constructed a microelectrode array sensor based on metalloporphyrin functionalized graphene, and realized ultra sensitive real-time monitoring of NO release from living cells. Jiang *et al*.^38^ developed a NO sensor based on hemin functionalized graphene FET for directly detecting NO in living cell with subnanomolar sensitivity. Keum *et al*.^39^ fabricated a polycarprolactone microneedle sensor at the end of an endomicroscope for both endomicroscopic imaging and NO biosensing of colon cancer, in which poly(3,4-ethylenedioxythiophene) and hemin molecules were coated on the microneedle surface. Therefore, the catalytic capability of porphyrin or hemin functionalized with nanomaterials can greatly enhance the detection sensitivity to NO. Although NO electrochemical sensors have been reported from various living cells^27, 40^, none of them has been utilized for real-time monitoring of NO in local acupoints. In our previous work^41, 42^, we have successfully fabricated nanomaterials modified acupuncture needle by electrochemical deposition of graphene or CNT on the surface of acupuncture needle, and explored electrochemical measurements for sensitive detection of dopamine and serotonin, respectively.

**Figure 1:**
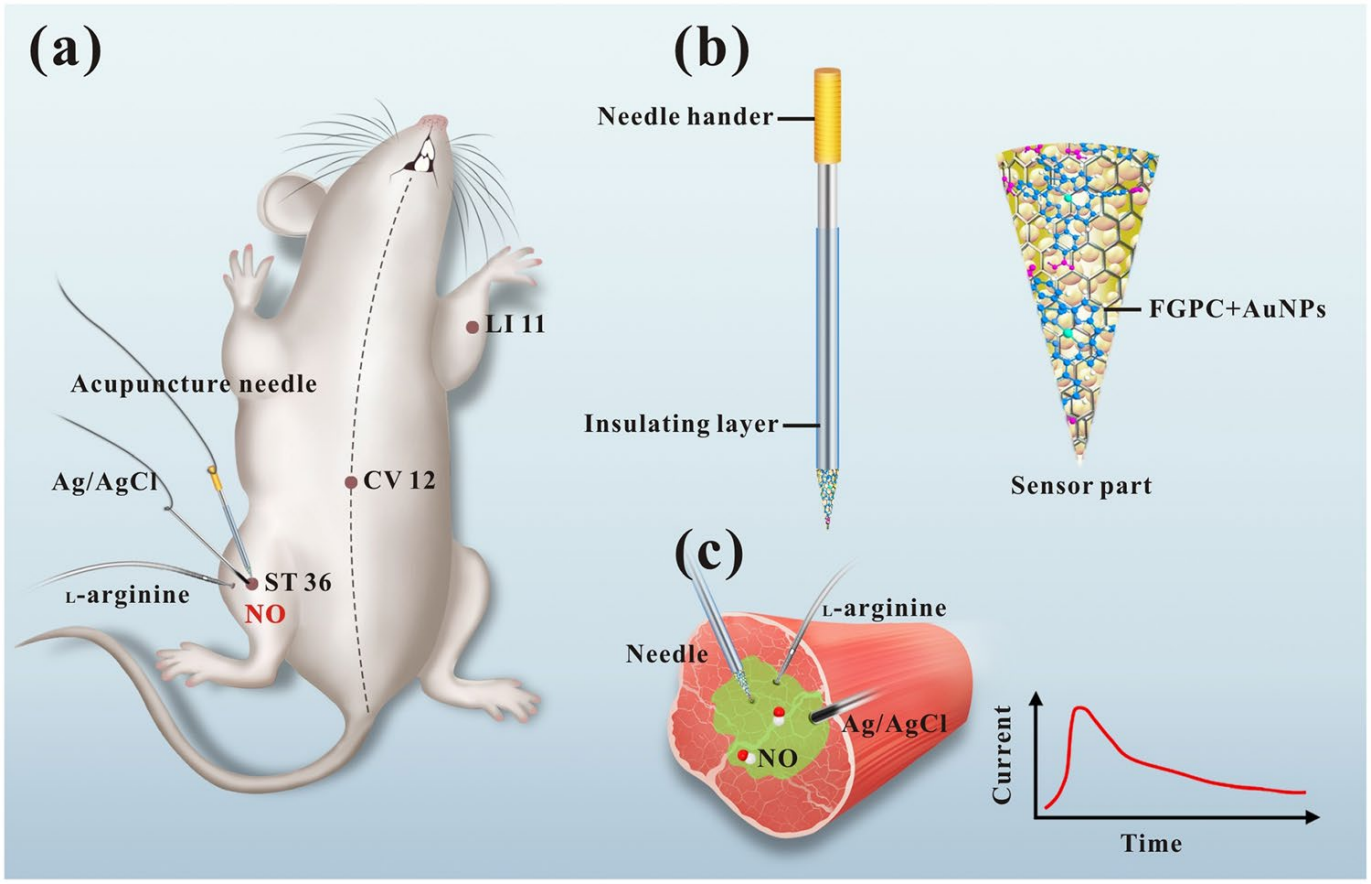
(**a**) Schematic illustration of a sensitive acupuncture needle microsensor for real-time monitoring of NO in acupoints of rats. (**b**) Schematic diagram of the FGPC/AuNPs/acupuncture needle. (**c**) Schematic diagram of real-time NO measurement in acupoint ST 36 stimulated by _L_-arginine.

In this report, we develop a unique acupuncture needle-based NO microsensor, capable of real-time monitoring NO in acupoint of rats by utilizing the superior electrical performance of graphene and the great catalytic property of porphyrin. As illustrated in Fig. 1, a gold film (presented as Au nanoparticles, AuNPs) is sprayed onto the tip of acupuncture needle by sputter coater. Subsequently, an iron-porphyrin functionalized graphene composite (FGPC) is prepared by hydrothermal synthesis, and the electrochemical deposition method is employed to deposit FGPC on the tip surface. The functionalized needle can successfully be applied in detecting NO with high sensitivity and satisfactory selectivity. Since NO can be generated in tissue by stimulated with _L_-arginine (_L_-Arg) in the presence of NO synthase (NOS) under physiological conditions, this functionalized acupuncture needle is applied for real-time monitoring of NO in different acupoints of rats, realizing *in vivo* detection of the signal molecule.

## Results and Discussion

### Characterization of the prepared FGPC and the FGPC functionalized needle

As is well known, Fe(III) meso-tetra (4-carboxyphenyl) porphyrin (FeTCP) can be conjugated to graphene with sp^2^ hybrid orbital to form FGPC. To verify the successful preparation of FGPC, UV-visible spectra were conducted. As shown in Fig. S1, characteristic absorption of GO at 227 nm, RGO at 263 nm, FeTCP at 416 nm could be observed, respectively. After FeTCP was conjugated with graphene through the π–π interaction^43^, the FGPC displayed two absorption bands at 263 nm and 430 nm, respectively, in which 263 nm was the RGO absorption band and 430 nm was caused by the Soret band of FeTCP with a red shift^44^.

To further prove that FGPC were synthesized successfully, X-ray photoelectron spectroscopy (XPS) was employed to explore the interaction between RGO and FeTCP. As shown in Fig. S2, the wide energy XPS spectra of FGPC (red line) and RGO (blue line) were obtained, respectively. Compared with RGO, the survey of FGPC exhibited higher ratios of C/O. In order to differentiate FGPC and RGO, narrow energy XPS spectra of Fe2p peak were analyzed. A remarkable Fe2p peak at 710 eV appeared from FGPC, while the peak was not seen from RGO. All these results indicate that GO has been reduced to RGO and FGPC were successfully obtained by further functionalizing RGO with FeTCP molecules.

After FGPC was successfully obtained, we started to fabricate FGPC functionalized acupuncture needle. Firstly, the gold film, presented as AuNPs, was deposited on the surface of acupuncture needle by ion sputtering. To obtain the FGPC layer, electrochemical deposition of FGPC is commonly employed in electrochemical biosensors, which is attractive due to its fast, inexpensive and green nature. Therefore, this preparation method was employed in this work to fabricate the FGPC/AuNPs/acupuncture needle, named as FGPC/AN. To verify the fabrication process, scanning electron microscope (SEM) was performed to show the surface morphology of acupuncture needles before and after nanomaterials modification. As seen in Fig. S3(a), the bare acupuncture needle (AN) had a smooth surface. After ion sputtering, the AuNPs were observed to be homogeneously distributed on the acupuncture needle (Fig. S3(b)). The AuNPs deposited onto the acupuncture needle could give rise to a large surface area and good electrochemical properties, providing a great interface for the next modification. After FGPC deposition, it was obviously seen that both PEDOT and FGPC covered the surface of AuNPs (Fig. S3(c)). As described earlier, the PEDOT can be served as glue water to enhance FGPC absorption on the tip surface of acupuncture needle. These SEM images demonstrate that FGPC have been successfully modified on the tip surface of needle as anticipated.

The electrochemical performances of the different nanomaterials modified needles were investigated by the cyclic voltammetry (CV) in K_3_Fe(CN)_6_ solutions. It was shown in Fig. S3(d) that bare AN had no distinct redox peak (black line), while the redox peak was observed on the nanomaterials modified needle. A significant response from Au/AN was obtained (blue line), which is caused by the good electrochemical properties of AuNPs. However, FGPC/AN showed larger current response than Au/AN. Firstly, FGPC could significantly enhance the conductivity of the electrode to facilitate the electron-transfer. Secondly, it has been reported that the electrical properties of electrode can be significantly improved by surface coating with PEDOT due to its outstanding conductivity^45–47^. Thirdly, after the needle was modified by nanomaterials, the surface area of needle could be enlarged and the electron-transfer could be facilitated^48, 49^. The excellent electrochemical properties of the FGPC/AN may be attributed to the synergistic effect of different nanomaterials.

The electrochemical impedance spectroscopy (EIS) experiments on the different ANs were further conducted. As seen in Fig. S4, bare AN showed a very large electron-transfer resistance. A smaller semicircular region was observed at Au/AN and FGPC/AN gave rise to the smallest semicircle. The resistance of bare AN (blue dots), Au/AN (red dots) and FGPC/AN (black dots) decreased, which is consistent with CV results in Fig. S3(d).

### Response of different needles to NO

**Figure 2:**
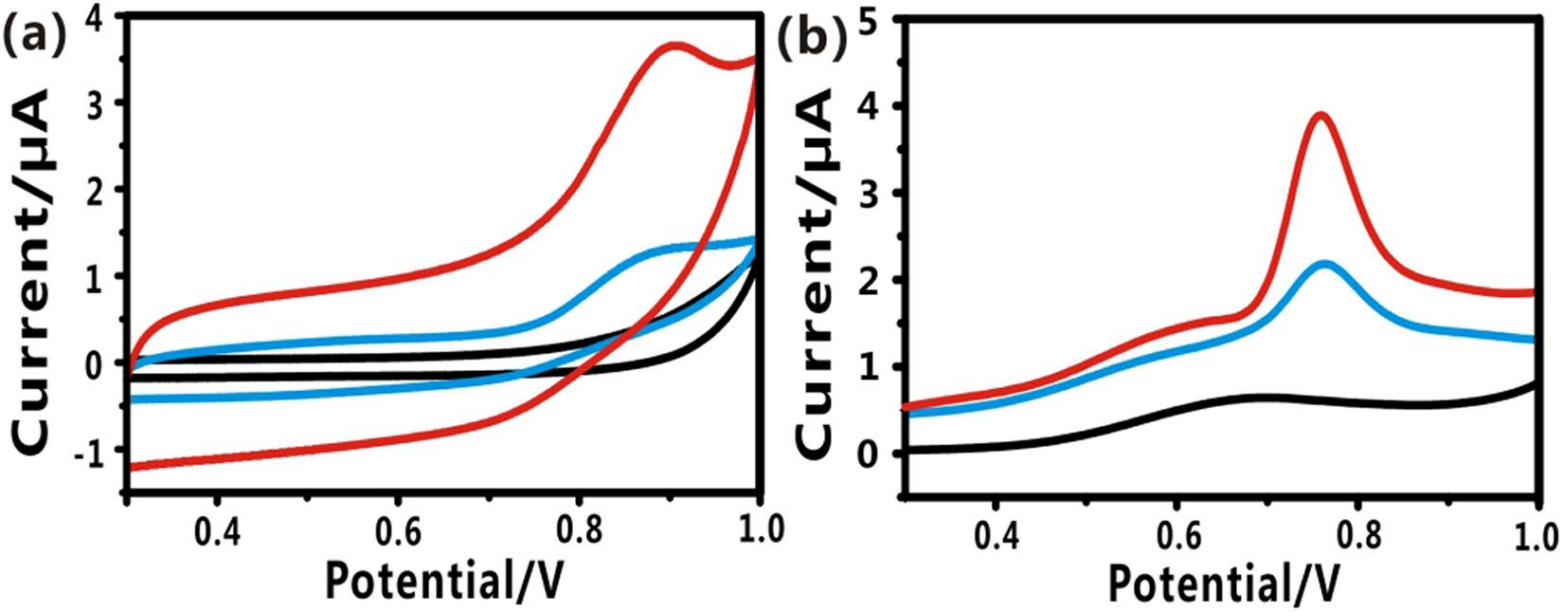
Electrochemical signals (**a**) CVs and (**b**) DPVs obtained at the different nanomaterials-modified acupuncture needle: bare AN (black line), Au/AN (blue line), FGPC/AN (red line) in deaerated PBS solution consisting of 18 μM NO.

The electrochemical performances of the needles, modified with different nanomaterials, were investigated by both CVs and DPVs in the presence of 18 μM NO. As shown in Fig. 2(a) and (b), FGPC/AN displayed excellent electrochemical behavior to NO, and showed peak currents at +0.88 V (CVs) and +0.78 V (DPVs), respectively (red line), while bare AN had negligible signal (black line). Au/AN also showed an obvious current response (blue line), which is ascribed to the fact that AuNPs possess the catalytic property to NO. However, it is worth noting that FGPC acted as the excellent catalytic capability and FGPC/AN showed larger current response than others. This attributes to the fact that FeTCP, simulating the enzyme active site^50^, could significantly enhance the catalytic capability to NO. Moreover, RGO can facilitate the fast electron-transfer between FeTCP and acupuncture needle. All above results demonstrate that the as-prepared acupuncture needle have good catalytic performance to NO.

### Selectivity and Sensitivity

**Figure 3:**
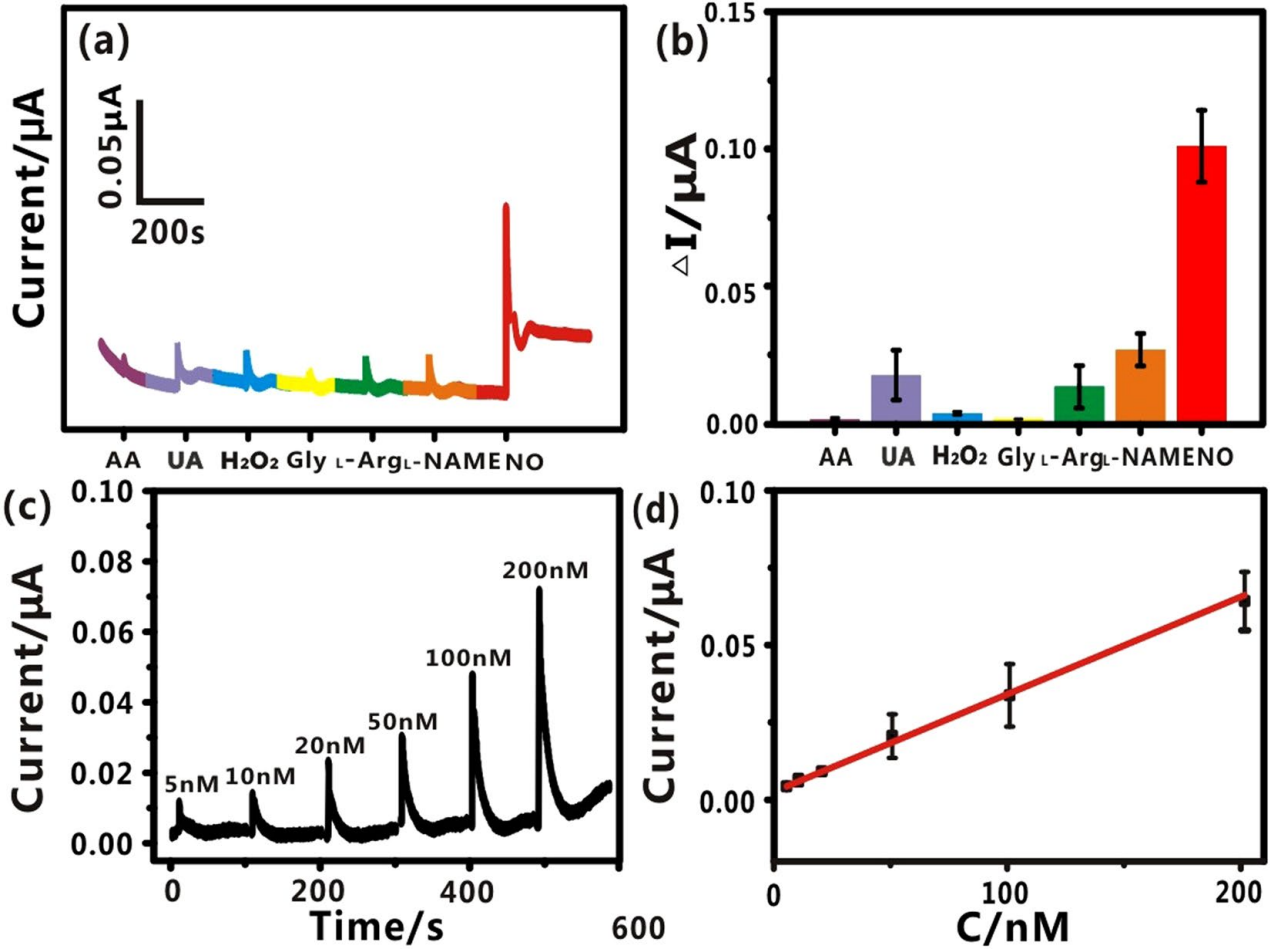
(**a**) Amperometric response of the FGPC/AN to interference substances in deaerated PBS solutions, in which all species were at the concentration of 1 mM. (**b**) Histogram of selective profile. (**c**) Amperometric response of FGPC/AN to the increased concentrations of NO in deaerated PBS solution at potential of +0.78 V (vs. Ag/AgCl). (**d**) The calibration curve of FGPC/AN to a series of NO concentrations. Ip(μA) = 0.0003C_NO_(nM) +0.0027, R^2^ = 0.9967. Error bars represent standard deviations of measurements (n = 3).

To investigate the selectivity of the functionalized needle, some common interfering species, such as ascorbic acid (AA), uric acid (UA), hydrogen peroxide (H_2_O_2_), glycine (Gly), _L_-arginine (_L_-Arg) and Nω-nitro-L-arginine methyl ester hydrochloride (_L_-NAME) were employed to interact with the FGPC modified needle, and the typical current responses of the needle to these substances were recorded. As displayed in Fig. 3(a), the current did not apparently change upon the addition of the interfering species, but remarkable current increases were observed upon addition of NO. To further illustrate the response difference, the current changes induced by the various substances were summarized in Fig. 3(b). It is very clear that FGPC/AN had a distinct response to NO, but a negligible response to other interfering substances. These results reveal that FGPC/AN has a satisfactory selectivity toward NO. The good selectivity might be attributed to the highly inherent specificity of the ferriporphyrin to electrocatalytic oxidation of NO, which is in good agreement with the previous report^40^.

To investigate the sensitivity of the FGPC modified needle, varying concentrations of NO in PBS from 5 nM to 200 nM were applied to the functionalized needle and current-time curves were recorded. Figure 3(c) shows typical amperometric response of the FGPC modified needle to successive addition of NO in deaerated PBS solution. It was obvious that the current was enhanced with the increasing concentrations of NO. The appearance of sharp peaks was probably caused by the diffusion. When NO was added to the solution, the concentration of NO increased dramatically on the electrode surface, causing the rising current. As the concentration of NO decreased, the current began to decay. The calibration curve of response of the FGPC modified needle to a series of NO concentrations was summarized in Fig. 3(d). The peak currents, assigned to the oxidation of NO, showed a linear response with the increasing NO concentrations in the range of 5 nM to 200 nM. The linear relationship was represented by Ip(μA) = 0.0003C_NO_(nM) +0.0027. The detection limit was calculated to be 3.2 nM with a signal-to-noise ratio of 3. It could be seen that the as-prepared FGPC/AN was sensitive and achieved a relative low detection limit.

To confirm the sensitivity of the fabricated FGPC/AN sensors toward NO, the comparative CV and DPV analyses were performed at various NO concentrations from 0 to 0.1 mM. Figure S5 presents CV and DPV responses to different concentrations of NO on the FGPC/AN, in which the current signal increased along with the increased NO concentrations. The CV and DPV results demonstrated that the FGPC/AN was able to detect NO as low as 10 nM (3 S/N), which is consistent with the I-T results.

The performance comparison of the fabricated FGPC/AN with other electrochemical sensors is listed in Table S1. Diab *et al*.^51^ electrodeposited a manganese porphyrin/polypyrrole films on Pt electrode for detection of NO with a limit of detection (LOD) of 100 nM was obtained. Zhang *et al*.^28^ developed a biosensor based on myoglobin adsorbed on multi-walled carbon nanotubes (MWNTs) and the LOD of 80 nM was obtained. Meanwhile, Zhang *et al*.^52^ reported ISO-NO Mark II NO meter (WPI) membrane-based carbon fibers for NO detection, and this nanosensor achieved detection limit down to 2 nM. To further challenge with high sensitivity, microarray electrode was used. Li *et al*.^32^ fabricated a TiC/C nanowire arrays microelectrode toward NO oxidation and achieved a LOD of 0.6 nM. Liu *et al*.^40^ reported an electrochemical method based on sensor array for NO detection and the low LOD of 55 pM was obtained. It is obvious that the sensitivity can be improved in the case that microelectrode and some molecules like porphyrin are employed. Compared with other electrochemical sensors for the determination of NO, the sensitivity of the as-prepared FGPC/AN is higher than that of the porphyrin/polypyrrole and CNT modified methods, but lower than that of carbon fiber and nanowire modified sensors. This is because the size of the needle tip surface is small (10 μm in diameter) and the modification of the nanomaterials is limited, thus influencing the detection sensitivity. Nevertheless, since NO is present at submicromolar level concentrations in the body^53^, the as-prepared needle is able to detection NO *in vivo*.

### Monitoring of NO release from living cells

**Figure 4:**
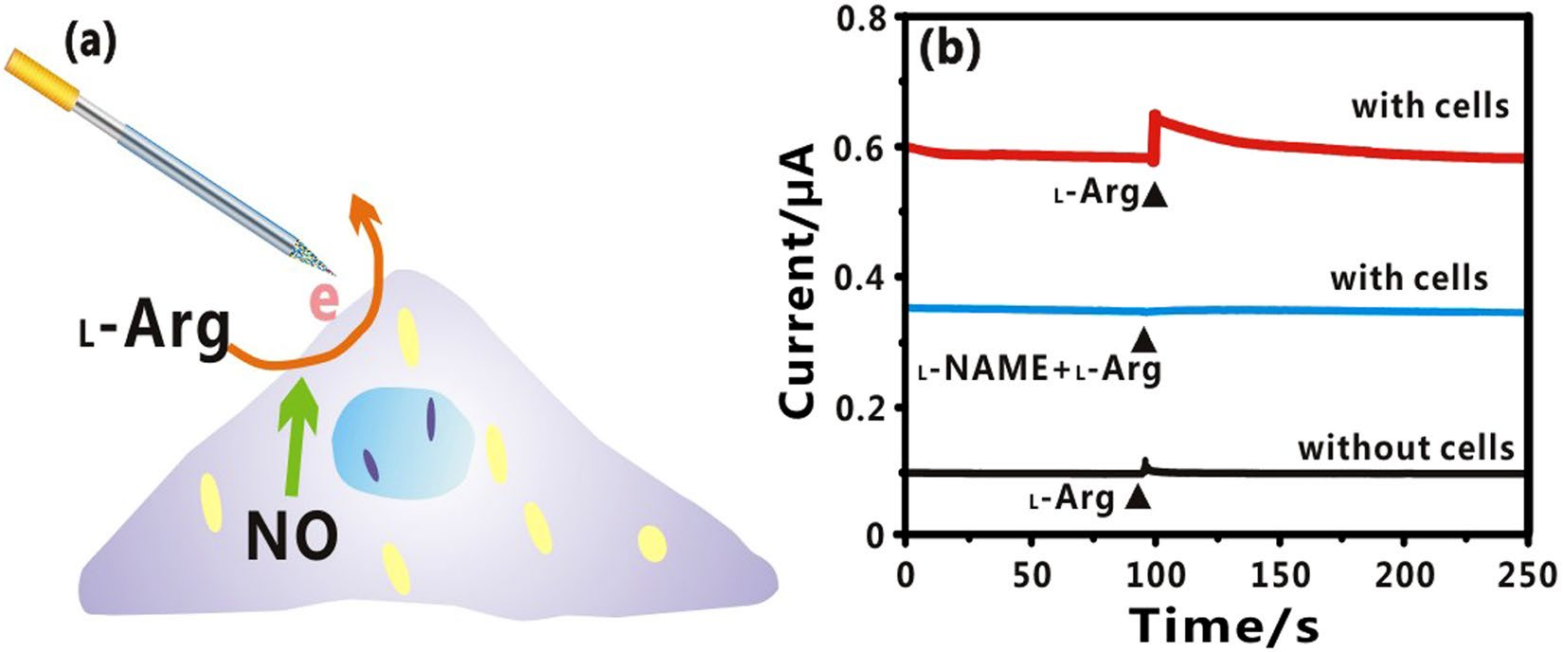
(**a**) Schematic illustration of the functionalized needle for real-time monitoring of NO release from living cell. (**b**) Amperometric response of the FGPC/AN to NO at cell level.

In order to test the sensor’s capability of detecting NO at cell level, real-time monitoring of NO release from human umbilical vein endothelial cells (HUVECs) was conducted. It is well known that HUVECs can generate NO when stimulated by _L_-Arg^27^. To realize the real-time monitoring of NO release from living cells, HUVECs were cultured on culture dish for 24 h. Prior to detection, the cell culture medium was removed, and supplemented with equivalent volume of PBS. Figure 4(a) illustrates the schematic diagram of real-time monitoring of NO release from living cell by using the FGPC modified needle. In this experiment, _L_-Arg was selected to stimulate cells for releasing NO, which could be enzymatically oxidated by NO synthase (NOS). On the contrary, _L_-NAME was used as a specific NOS inhibitor to block NO release.

The amperometric responses of the FGPC modified needle to NO in different conditions were shown in Fig. 4(b). When _L_-Arg was introduced into the cells, the current increased remarkably and then started to stabilize slowly (red line). On the contrary, introduction of _L_-NAME (10 mM) diminished the current change (blue line), owing to the specific inhibiting behavior of _L_-NAME toward NO release. A control experiment was conducted to verify that the increasing current was caused by the NO release from HUVECs. When the same concentration of _L_-Arg was added into the detection chamber without cells, no obvious current response was observed (black line). All above experiments demonstrate that the functionalized needle is capable of monitoring NO in real time at cell level.

The toxicity test was performed on the different sensing needles by using MTT assay. Briefly, the tip surface with the same length on bare AN, Au/AN and FGPC/AN was cut, respectively. And the cut tip was then cultured separately with HUVECs. Meanwhile, the control group without adding any needle was performed. After 24 h incubation, MTT solution was applied, and the resultant supernatants were assayed by a spectrophotometer (Bio-RAD, USA). The results from the MTT assay are shown in Fig. S6. It can be seen that the normalized viabilities of bare AN, Au/AN and FGPC/AN remained as high as 92%, 94%, 95%, respectively, indicating that the cytotoxic of the sensing needle is negligible.

### Real-time monitoring of NO release *in vivo*

The stability of the prepared needle was investigated by both *in vitro* and *in vivo* experiments. After kept in 1 M KCl solution for 7 days, the needles were immersed into NO solution and scanned (Fig. S7(a)). Electrochemical CV results showed that the needle remained over 83% of its initial current. Furthermore, stability regarding *in vivo* experiments was also conducted. After the modified needle was inserted into acupoints, a potential of +0.78 V was applied. As shown in Fig. S7(b), we could see that the amperometric response decreased sharply and kept quite stable as a function of time (3000 s). After *in vivo* amperometric measurement, the needle was examined by SEM in order to verify if the modified layer remained on the tip surface of the needle. As clearly seen in Fig. S8, FGPC still covered on the needle body. All these results indicate that FGPC/AN can be used for *in vivo* experiment with excellent stability.

To verify whether the functionalized acupuncture needle has the capability of real-time monitoring NO *in vivo*, NO standard solution (1 μM) was pumped into the acupoint Zusanli (ST36) and the current was measured *in vivo* as a proof-of-concept experiment. The experimental procedure was carried out as follows: male rats were anesthetized with chloral hydrate and then fastened to an animal operating table before *in vivo* measurement. FGPC/AN was inserted vertically at ST36, and the reference electrode was placed nearby. The syringe needle was used to microinfuse the prepared NO solutions into the corresponding positions of rats. After the NO solution was injected, it could be seen that a significant current response appeared, yet the injected PBS solution as control did not provoke any change in the baseline current except for some tiny disturbance (Fig. S9), suggesting that the as-prepared FGPC/AN was capable of detecting NO *in vivo*.

**Figure 5:**
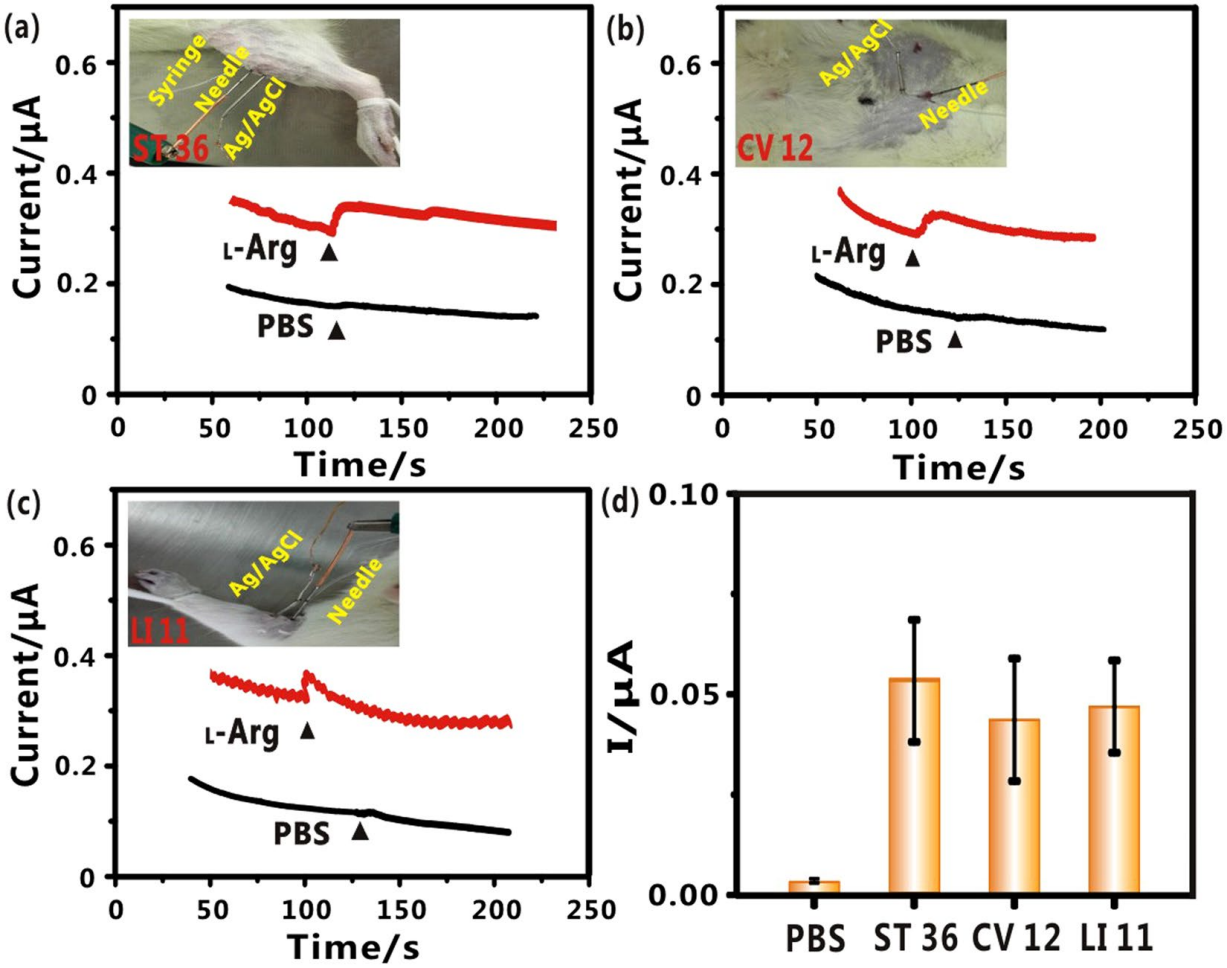
Real-time monitoring of NO in different acupoints by the FGPC modified needle: (**a**) Amperometric response of FGPC/AN to NO stimulated by _L_-Arg (red line) and PBS (black line) in ST36. Inset: Optical image of *in vivo* measurement. (**b**) Amperometric response of FGPC/AN to NO stimulated by _L_-Arg (red line) and PBS (black line) in LI11. Inset: Optical image of *in vivo* measurement. (**c**) Amperometric response of FGPC/AN to NO stimulated by _L_-Arg (red line) and PBS (black line) in CV12. Inset: Optical image of *in vivo* measurement. (**d**) Histogram of the corresponding amperometric response of the needle to NO release from different acupoints. Error bars represent standard deviations of measurements (n = 8).

On the basis of the excellent properties of the FGPC modified needle, the needle was then inserted into the different acupoints to realize real-time monitoring of NO dynamic change while stimulated by _L_-Arg. The typical acupoints of ST36, Zhongwan (CV12) and Quchi (LI11) were selected, respectively. All those acupoints could be easily identified on the body surface and were used to promote blood flow of local circulation and treat cardiovascular disease^15^. As shown in Fig. 5(a), after FGPC/AN was inserted into the acupoint of ST36, followed by _L_-Arg stimulation, an apparent current increase was observed. As a control, if stimulated by PBS solution instead of _L_-Arg, hardly current change was seen, indicating that FGPC/AN could realize the real-time monitoring of NO in acupoints when stimulated by _L_-Arg. Then, real-time monitoring of NO in other acupoints was also carried out, and the similar results were obtained, as exhibited in Fig. 5(b) and (c). When FGPC/AN was inserted into the acupoints of CV12 and LI11, obvious current changes evoked by _L_-Arg appeared, respectively. Similar to the above-mentioned control experiments, negligible current change was found when PBS replacing _L_-Arg was injected. The corresponding amperometric response of the functionalized needle to NO release from different acupoints was summarized in Fig. 5(d). It was found that similarly obvious response from the three different acupoints were observed (each measurement was repeated 8 times, and 8 groups of Wistar rats were employed). The actual NO concentration detected in acupoints could be semi-quantitively obtained by the working curve in Fig. 3(d). Based on the current change generated in ST 36 in Fig. 5, the NO concentration was calculated to be about 150 nM, which is close to physiological NO level (submicromolar level)^53^. Now that NO dynamic change in acupoint was detected, the NO level in non-acupoint for further comparison was investigated. In the experiments, the non-acupoints were selected at about 2 cm beside CV12 and lateral of stomach meridian in abdomen. As shown in Fig. S10, there was a slight current change when FGPC/AN was inserted into non-acupoint, followed by stimulation of the same concentration of _L_-Arg. All these results indicate that a direct real-time measurement of NO in acupoints have been realized, demonstrating the needle’s superior detection capability *in vivo*.

Generally speaking, the level of NO in acupoints is related to the expression of NOS in meridian skin regions. Higher NOS activity in meridians and acupoints has been found in rats. Our results also show that the NO level in acupoint is much higher than that in non-acuponts, which is consistent with the reported literature^16, 17^. Until now, the accurate concentration of NO in acupoints has not been reported yet, meaning that the relationship between amount of NO detected in acupoints and what kind of molecular events occurred in acupoints is still unknown. It is certain that we will continue to conduct further in-depth study in the next work. Anyway, this work reported for the first time that the functionalized acupuncture needle could be directly probed into rat body for real-time monitoring of the signal molecules in acupoints, which is a big progress compared with the previous work. This study essentially opens a new process for *in vivo* electrochemical biosensing, which is envisaged to be of great importance in understanding molecular basis and underlying the mechanism of acupuncture.

## Conclusion

In conclusion, we have developed a functional acupuncture needle based on ferroporphyrin and graphene composites for real-time and *in vivo* monitoring of NO in acupoints with superior performance. The FGPC functionalized acupuncture needle exhibited efficient electrocatalysis, high sensitivity and good selectivity toward NO, making it capable of realizing real-time monitoring of NO in complex environments including cell measurement and *in vivo* detection. This work offers a simple and reliable method for preparation of nanomaterials functionalized acupuncture needle, serving as a microsensor for real-time and *in vivo* monitoring of NO in acupoints. It is believed that such a sensing platform will provide a significant technical support and new broad vision in the study of acupuncture, and play a positive role in promoting the development of traditional Chinese acupuncture and moxibustion medicine.

## Methods

### Reagents and materials

Acupuncture needles, made of Ni-Cr alloy, were purchased from Suzhou medical supplies factory Co. Ltd. (Suzhou, China). Graphene oxide (GO) was obtained from Xian Feng Nano Technology Co. Ltd. (Nanjing, China). Hydrazine solution (85%) and ammonia solution (25 wt%) were obtained from Aladdin Biological Technology Co. Ltd. (Shanghai, China). Epoxy resin was purchased from Liyang Kangda Chemical Co. Ltd. (Nanjing, China). Fe(III) meso-tetra (4-carboxyphenyl) porphyrin (FeTCP, cas:55266-17-6) was purchased from Frontier Scientific, Inc. (Beijing, China).

The cell culture medium RPMI 1640, L-glutamine, HEPES, trypsin and glycine (Gly) for human umbilical vein endothelial cells (HUVECs) culture were purchased from GIBCO (Carlsbad, CA, USA). 3,4-ethylenedioxythiophene (EDOT), N, N-dimethylformamide (DMF), _L_-arginine (_L_-Arg), Nω-nitro-L-arginine methyl ester hydrochloride (_L_-NAME) and methyl thiazolyl tetrazolium (MTT) were purchased from Sigma (St. Louis, MO, USA). The HUVECs lines were obtained from Xiangya hospital (Changsha, China). The other reagents, like ascorbic acid (AA), uric acid (UA), dimethylsulfoxide (DMSO) and hydrogen peroxide (H_2_O_2_) were purchased from Sinopharm Chemical Reagent Co. Ltd. (Shanghai, China). All reagents unless specified were of analytical grade and were used as received without further purification. Ultrapure water was prepared by Milli-Q System (Billerica, MA, USA).

### Apparatus and characterization

Electrochemical measurements were performed on CHI660D electrochemical workstation (Chenhua Co. Ltd., Shanghai, China). A conventional three-electrode system, which was composed of a bare or modified acupuncture needle as the working electrode, an Ag/AgCl electrode as the reference electrode and a platinum wire as the auxiliary electrode, was employed throughout the experiment. During the *in vivo* measurements, the solutions (PBS or _L_-Arg) were delivered by a LSP02-1B microinjection pump (Baoding Lange, Baoding, China). UV-vis absorption spectra were recorded on a UV-2550 spectrophotometer (Shimadzu, Japan). X-ray photoelectron spectroscopy (XPS) measurement was carried out on an XSAM800 photoelectron spectrometer (Kratos, UK). Scanning electron microscopy (SEM) images were obtained on a field-emission scanning electron microscope (Zeiss, Germany). UV-visible spectra, XPS and SEM were used to characterize the structure of FGPC. All measurements were carried out at a room temperature.

### Preparation of FGPC

FGPC was prepared according to the literature^36, 54, 55^ with a small modification. Briefly, 8 mg of GO was initially added into 6 mL of ultrapure water, and the resultant mixture was vigorously sonicated for 1 h at room temperature to obtain a homogeneous suspension. After that, 10 mg of FeTCP, dispersed in 2 mL of DMF/water (4:1, v/v), was dropped into the suspension. The resulting mixture was further sonicated for 1 h. During this process, FeTCP was assembled onto the GO by the hydrophobic interactions and π-π stacking. The porphyrin-functionalized graphene oxide (designated as FeTCP/GO) was obtained through centrifugation (10,000 g, 30 min), and re-dispersed into 8 mL of ultrapure water. Next, 4 μL of hydrazine solution and 50 μL of ammonia solution were added to the above solution and the mixture was put in a water bath (95 °C) for 1 h under agitation. The resulting product was subsequently filtered through a Nylon membrane (0.15 μm) and thoroughly washed with water until the pH of the filtrate reached 7.0. The as-prepared nanocomposite was ultrasonically dispersed in ultrapure water to obtain a FGPC solution (0.1 mg/mL).

### Preparation of NO solution

The NO stock solution (1.8 mmol/L) was prepared by bubbling 15 mL of deaerated aseptic 1 × PBS solutions with purified NO gas. Different concentrations of NO standard solutions were obtained by diluting the stock solution with 1 × PBS gradually.

### Fabrication of FGPC on acupuncture needles

Prior to FGPC modification, a gold film was sprayed onto the acupuncture needle by JFC-1600 sputter coater (JEOL, Japan) with current 0.03 mA, time 30 s for the purpose of an ideal modification at the next step and enhancement of conductivity. Then an insulated epoxy resin layer was coated on the needle body, just leaving the tip of active surface for FGPC modification. In order to obtain the modified layer preferably, EDOT was added in the FGPC solution which was served as glue water, as discussed in our previous work^42^. The FGPC modified acupuncture needle was then achieved by electrochemical methods^56, 57^. The electrochemical deposition was performed in the above-mentioned mixture on the electrochemical station (−0.9 V~1.25 V, 20 m V/s). After deposition, the FGPC modified acupuncture needle was got and kept in 0.1 M KCl for the further use. Both CV and EIS were used to characterize the performance of sensing needles in aqueous solution consisting of 5 mmol/L of K_3_[Fe(CN)_6_] and 0.1 mol/L of KCl.

### Electrochemical detection of NO

To carry out the electrochemical detection, cyclic voltammograms (CVs, voltage range: 0.3 to 1.0 V, scan rate: 100 mV/s), differential pulse voltammetry (DPV, 0.3~1.0 V, step potential, 0.004 V, modulation amplitude, 40 mV, pulse width, 0.05 s, pulse period, 0.2 s) and amperometric curves were performed in 0.001 M PBS. The current-time curves were performed at +0.78 V vs Ag/AgCl (obtained from the DPV anodic current). All the current response data were obtained under magnetic stirring until the testing solution was achieved to be homogeneous. PBS solution was deoxygenated by bubbling pure nitrogen for at least 5 min and then the NO solution was added into this PBS, after which nitrogen atmosphere was maintained during the experiments.

### Detection of NO Release from HUVECs

HUVECs were routinely cultured using RPMI 1640 culture medium in a culture flask at 37 °C in a humidified incubator (95% air with 5% CO_2_). For NO detection, the cells were then digested and transferred to the 1 × 1 cm glass, after which the glass was placed into a glass culture dish in incubator. After 24 h, 2 mL of culture medium was replaced by equal volume of aseptic PBS. A three-electrode system was employed, including the FGPC modified acupuncture needle, Ag/AgCl reference electrode and Pt counter electrode. The amperometric detection was carried out by applying a constant potential of +0.78 V, and the NO release was evoked by _L_-Arg (3 mM).

### *In vitro* cytotoxicity test

HUVECs were routinely cultured using RPMI 1640 culture medium and used for *in vitro* cytotoxicity test of ANs. For cell MTT assay, 96 well plates were seeded with 6 × 10^4^ HUVECs/ml in 100 μL of cell culture medium and incubated under cell culture conditions for 24 h. Thereafter, the tip surfaces of bare AN, Au/AN and FGPC/AN with the same length (4 mm in length) were cut and added to different wells, respectively. After cells were further incubated with the various ANs for 24 h, 100 μL of MTT solution (1 mg/mL) was added into each well and incubated for 4 h. Finally, 150 μL of DMSO were added to the plates, shaked for 5 min. Finally the resultant supernatants were assayed at a wavelength of 520 nm by a spectrophotometer (Bio-RAD, USA).

### *In vivo* measurements in acupoints

Wistar rats (250–350 g, Male, SPF, NO. 42000600013006) were purchased from the Experimental Animal Center of Centers for Disease Control and Prevention of Hubei Provincial. The rats were housed on a light-dark schedule (12/12 h) with food and water at random. After being generally anesthetized with 400 mg/mL chloral hydrate, the rats were positioned onto animal operating table. The FGPC modified needles were inserted vertically with a depth of 3–5 mm^58, 59^ in the typical acupoints of Zusanli (ST36), Zhongwan (CV12) and Quchi (LI11)^60^. In order to prevent the modified layer from falling off, a guide cannula was inserted into the acupoints. Then the needle was inserted into the cannula. The reference electrode was positioned nearby the needle in the same way. The length of the exposed needle *in vivo* was about 4 mm. The distance among the sensor, injector and reference electrode was about 8 mm. The syringe needle was used to microinfuse the solutions into the acupoints of rats (250 μM/min). After that, the real-time monitoring experiment was performed and the amperometric detection was conducted. The injection of PBS into the acupoints was used as control, and the injection of 3 mM _L_-Arg into the acupoints, was employed for the purpose of simulating an endogenous release of NO. All experiments on animals were carried out in accordance with the approved guidelines. The study was approved by the Animal Research Committee of Hubei University of Chinese Medicine.

## Electronic supplementary material

Supplementary Information
