# Supplementary material for: A sensitive acupuncture needle microsensor for real-time monitoring of nitric oxide in acupoints of rats
Source: Sci Rep. 2017 Jul 25;7:6446. doi: 10.1038/s41598-017-06657-3 (PMC5527006; doi:10.1038/s41598-017-06657-3)
Supplement: Supplementary file 1 — Supplementary Information [file 41598_2017_6657_MOESM1_ESM.doc]

**Supporting Information for**

**A sensitive acupuncture needle microsensor for real-time monitoring of nitric oxide** **in acupoints of rats**

Lina Tang^1,3^, *^§^*, Yutao Li^1,3^, *^§^*, Hui Xie^1^, Qing Shu^2^, Fan Yang^1^,Yan-ling Liu^4^, Fengxia Liang^2,3^, Hua Wang^2,3^,Weihua Huang^4^, Guo-Jun Zhang^*1,3^

^1^ School of Laboratory Medicine, Hubei University of Chinese Medicine

1 Huangjia Lake West Road, Wuhan 430065, China.

^2^Department of Acupuncture and Moxibustion, Hubei University of Chinese Medicine, 1 Huangjia Lake West Road, Wuhan 430065, China.

^3^Hubei Provincial Collaborative Innovation Center of Preventive Treatment,

1 Huangjia Lake West Road, Wuhan 430065, China.

^4^Key Laboratory of Analytical Chemistry for Biology and Medicine, Ministry of Education, College of Chemistry and Molecular Sciences, Wuhan University,

4. Bayi Road, Wuhan 430072, China

*^§^*These authors contributed equally to this work

*Corresponding author: Tel: +86-27-68890259, Fax: +86-27-68890259

Email: [zhanggj@hbtcm.edu.cn](mailto:zhanggj@hbtcm.edu.cn)


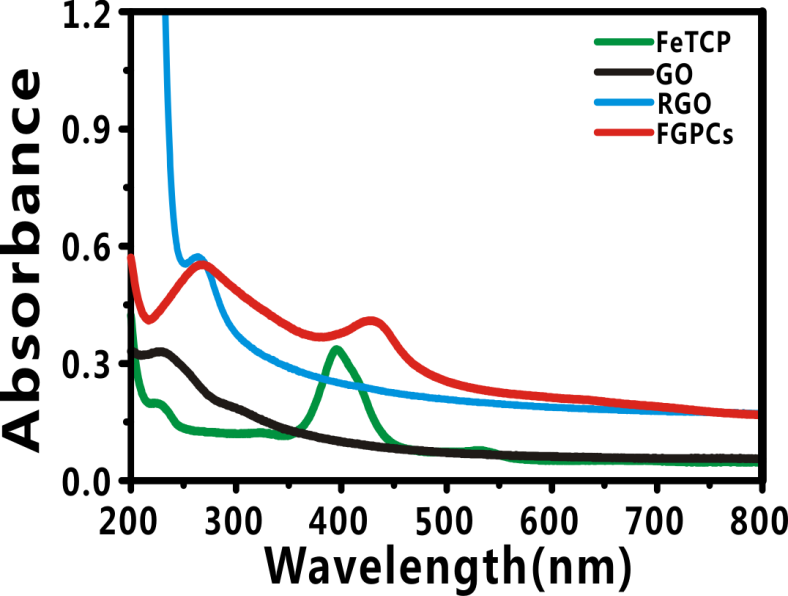


**Figure S1.** UV-visible spectra of FeTCP solution (green line), GO suspension (black line), RGO (blue line), and FGPC suspension (red line).


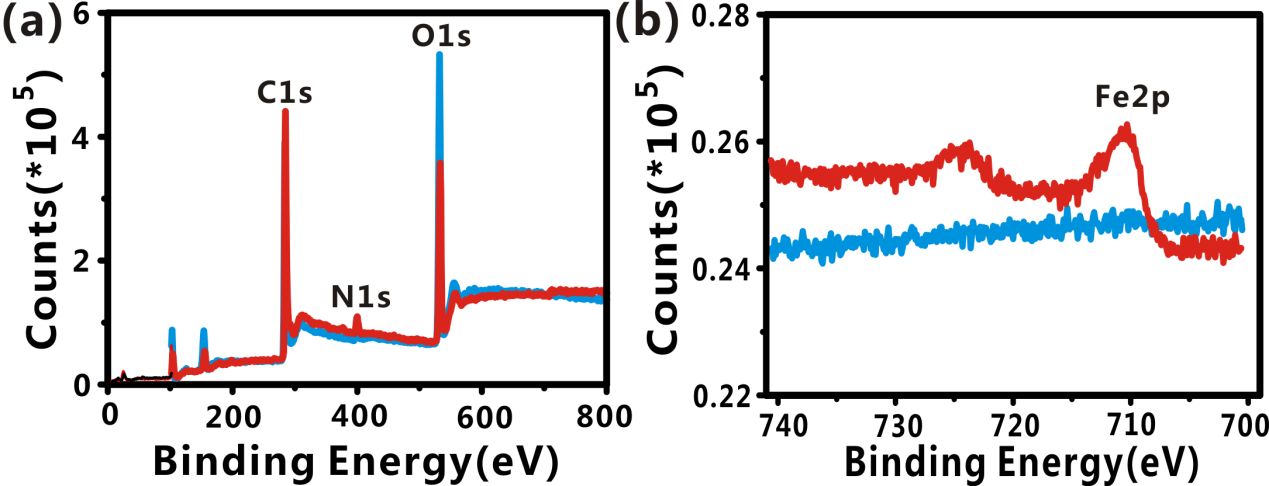


**Figure S2.** (a) XPS data （wide survey） for RGO (blue line) and FGPC (red line). (b) Narrow survey of Fe2p contributed by RGO (blue line) and FGPC (red line), respectively.


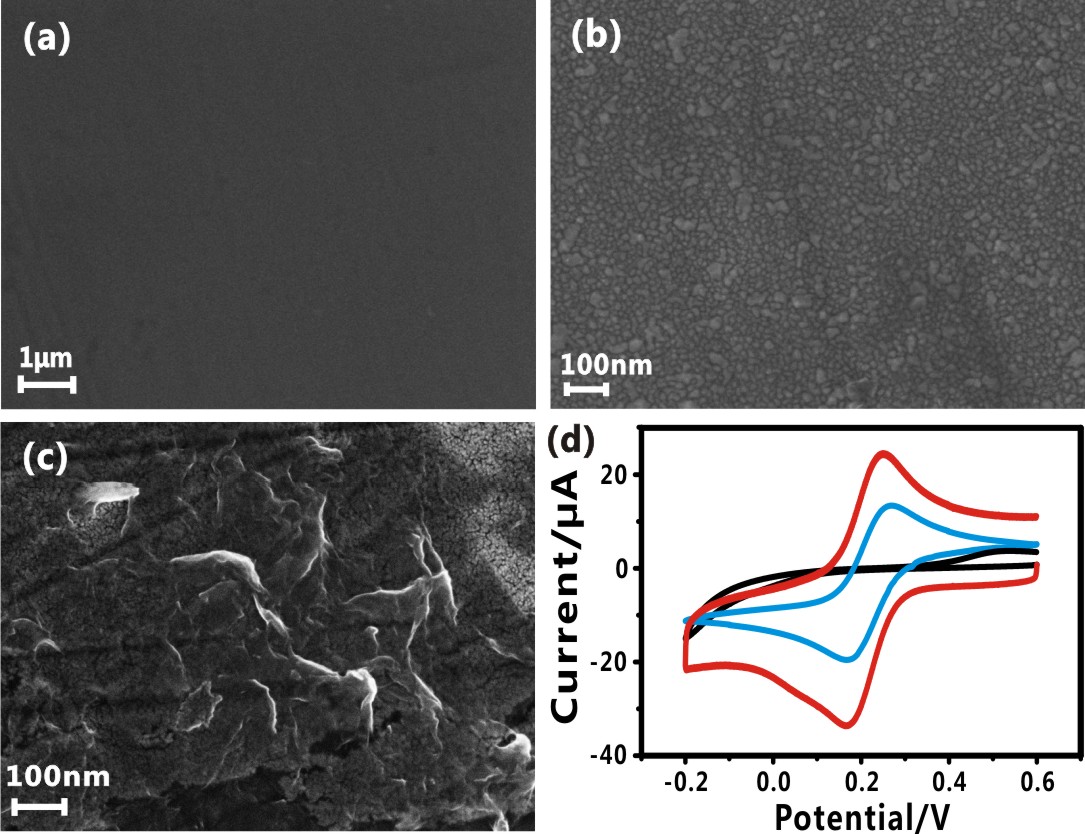


**Figure S3.** (a) SEM images of (a) bare acupuncture needle (AN), (b) AuNPs/acupuncture needle (Au/AN), (c) FGPC/AuNPs/acupuncture needle (FGPC/AN). (d) CVs obtained at the different nanomaterial-modified acupuncture needle: bare AN (black line), Au/AN (blue line), FGPC/AN (red line) in aqueous solution consisting of 5 mmol/L of K_3_[Fe(CN)_6_] and 0.1 mol/L of KCl. The voltage range: -0.2 to 0.6 V; scan rate: 100 mV/s.


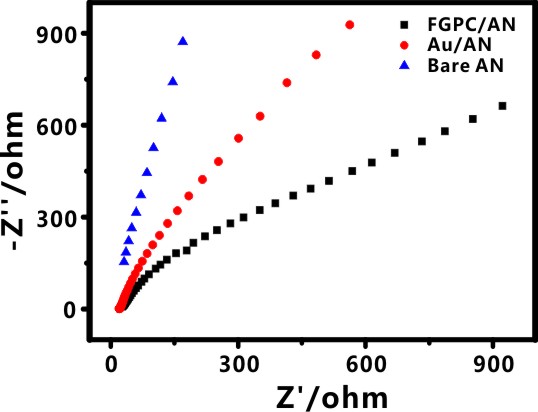


**Figure S4.** EIS obtained at the different nanomaterial-modified acupuncture needles: bare AN (blue dots), Au/AN (red dots), FGPC/AN (black dots) in the presence of 5 mmol/L of K_3_[Fe(CN)_6_] and 0.1 mol/L of KCl.


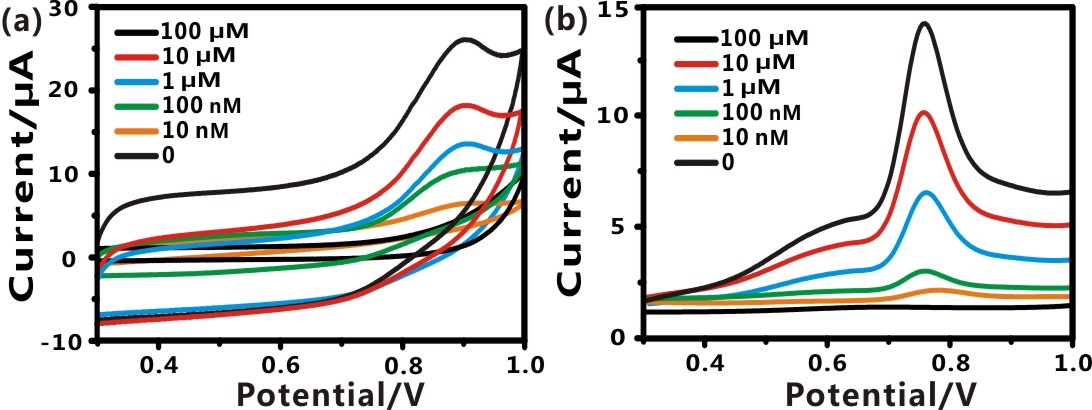
**Figure S5.** (a) The CVs versus increasing NO concentrations. (b) The DPVs versus increasing NO concentrations of 0, 10 nM, 100nM, 1μM, 10μM and 100μM (from bottom to top).

**
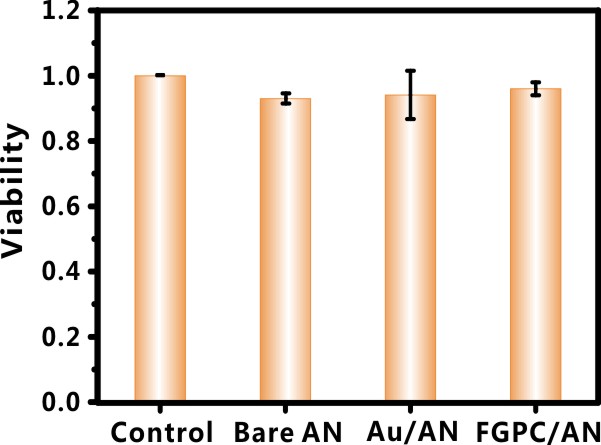
**

**Figure S6.** In vitro cytotoxicity test. Normalized viability of HUVECs cultured with the different sensing needles for 24 h. Error bars represent standard deviations of measurements (n = 5).


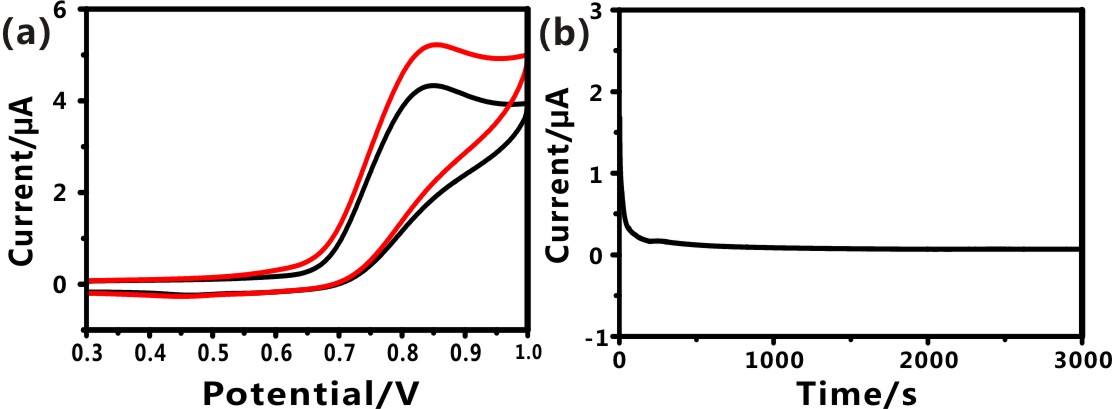


**Figure S7.** (a) CVs of FGPC/AN in aqueous solution of NO (1mM) before (red line) and after (black line) stored in KCl solution for 7 days. (b) *In vivo* amperometric response recorded with FGPC/AN.


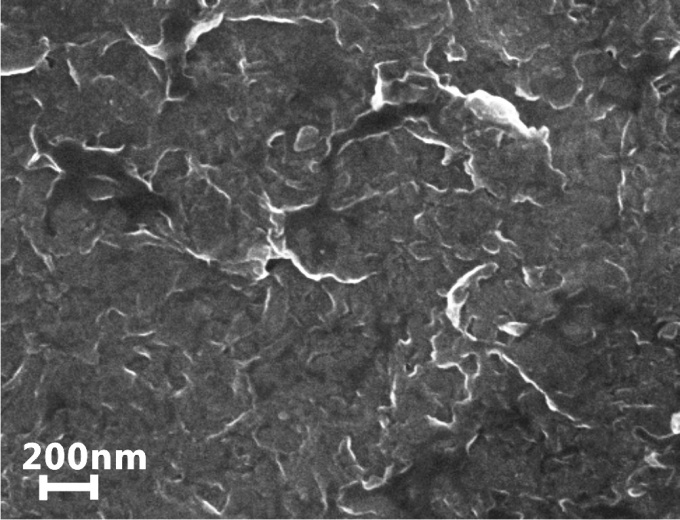


**Figure S8.** The SEM image of FGPC/AN after inserted in acupoint.


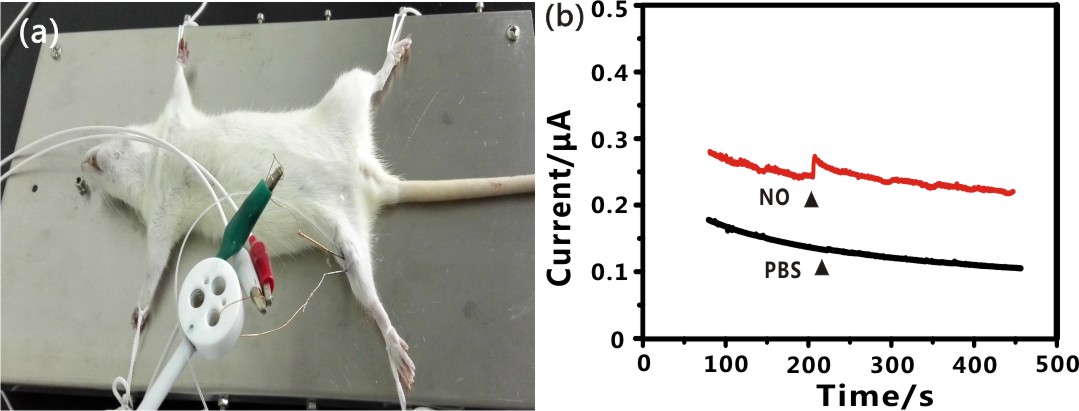


**Figure S9.** (a) Optical image of *in vivo* measurement setup. (b) Amperometric response of FGPC/AN to NO (red line) and PBS (black line), respectively, that was pumped into acupoint.


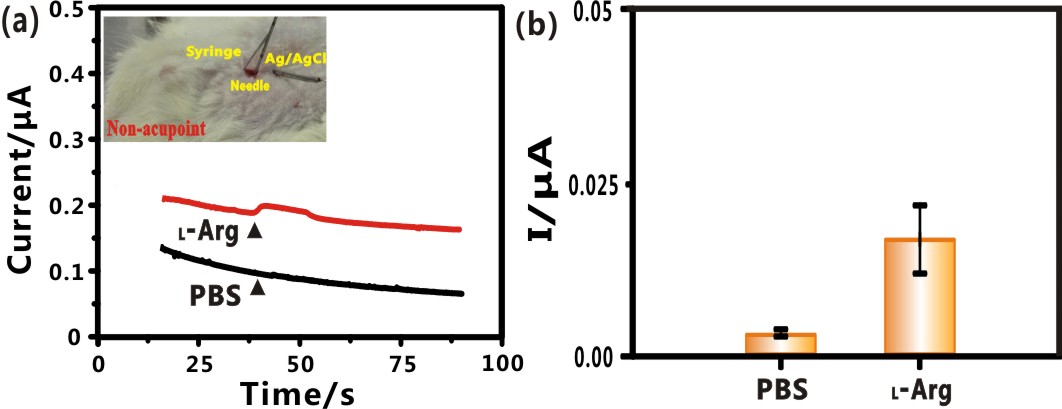


**Figure S10.** (a) Amperometric response of FGPC/AN to NO stimulated by _L_-Arg (red line) and PBS (black line) in non-acupoint. Inset: Optical image of *in vivo* NO measurement from non-acupoint. (b) Histogram of the corresponding amperometric response of the needle to NO release from non-acupoint. Error bars represent standard deviations of measurements (n = 8).

**Table S1.** Sensitivity comparison of the proposed FGPCs/AN with other electrochemical sensors

| Basement | Modified materials | Detection limit(nM) | Linear range(nM) | Method | References |
| --- | --- | --- | --- | --- | --- |
| Glassy carbon | Myoglobin-  MWNT^a^ | 80 | 200- 4.0×10^4^ | Amperometry | [28] |
| Microelectrode arrays | TiC/C NW^b^ | 0.6 | 5-1000 | Amperometry | [32] |
| Glassy carbon | MWNT^c^ | 80 | 200- 1.5×10^5^ | Amperometry | [33] |
| Micro-electrochemical sensor array | FGHNs^d^ | 0.055 | 5-100 | Amperometry | [40] |
| Pt microdisk | Nafion/porphyrin/polypyrrole films | 100 | 5×10^3^-1.20×10^5^ | DPV | [51] |
| Carbon fiber | WPI membrane/Nafion | 2 | 10-1000 | Amperometry | [52] |
| Acupuncture needle | FGPCS/AuNPs | 3.2 | 5-200 | Amperometry | This work |

^a^ Myoglobin adsorbed on multi-walled carbon nanotubes

^b^ TiC/C nanowire arrays

^c^ multi-walled carbon nanotubes

^d^metalloporphyrin and 3-aminophenylboronic acid (APBA) co-functionalized reduced graphene oxide
